# Diffusion-Weighted Imaging Findings of Ischemic Spinal Injury in a Chondrodystrophic Dog With Fibrocartilaginous Embolism

**DOI:** 10.3389/fvets.2020.598792

**Published:** 2020-12-10

**Authors:** Taesik Yun, Kang-Il Lee, Yoonhoi Koo, Hakhyun Kim, Dongwoo Chang, Chulhyun Lee, Mhan-Pyo Yang, Byeong-Teck Kang

**Affiliations:** ^1^Laboratory of Veterinary Internal Medicine, College of Veterinary Medicine, Chungbuk National University, Cheongju, South Korea; ^2^Section of Veterinary Medical Imaging, Veterinary Teaching Hospital, College of Veterinary Medicine, Chungbuk National University, Cheongju, South Korea; ^3^Division of Convergence Biotechnology, Korea Basic Science Institute, Ochang, South Korea

**Keywords:** apparent diffusion coefficient, diffusion-weighted imaging, dog, fibrocartilaginous embolism, high-field MRI

## Abstract

A 9-year-old, intact male Shih Tzu dog presented with systemic weakness and peracute onset of tetraplegia. Tetraplegia with lower motor neuron signs was noted upon neurological examination. Diseases that cause acute flaccid tetraparesis, such as acute fulminating myasthenia gravis, polyradiculoneuritis, tick paralysis, and botulism, were ruled out based on the medical history, normal electrophysiological tests, and non-response to the neostigmine challenging test. Initial 0.3-Tesla (T) magnetic resonance imaging (MRI) findings included sharply demarcated intramedullary lesions at the C3-C6 level, mainly involving gray matter, which appeared hypo- to iso- intense on T1-weighted images (WIs), and hyperintense on T2-WIs and fluid-attenuated inversion recovery images. There was no enhancement on post-contrast T1-WIs. Neutrophilic pleocytosis was observed in the cerebrospinal fluid analysis. No clinical responses were observed for the treatment of non-infectious myelitis with an immunosuppressive dosage of prednisolone. A follow-up 3-T MRI 6 days later demonstrated hyperintensity on diffusion-WI (DWI) and a decreased apparent diffusion coefficient (ADC) value (0.54 × 10^−3^ mm^2^/s) of the spinal lesions. Through histological examination, a fibrocartilaginous embolism was definitively confirmed. This is the first report to describe an ischemic spinal injury visualized by DWI and ADC mapping with high-field MRI in a chondrodystrophic dog diagnosed with a fibrocartilaginous embolism.

## Introduction

Fibrocartilaginous embolism (FCE), first reported in dogs in 1973, is an acute ischemic myelopathy caused by fibrocartilaginous materials that block vasculature of the spinal cord (1). The emboli, which are histochemically the same as the nucleus pulposus of intervertebral disks, induce ischemic myelopathy.

FCE is definitively diagnosed by histopathological examination of the spinal cord. Therefore, antemortem diagnosis of FCE is based on history, signalments, clinical signs, and exclusion of other causes using survey radiographs, cerebrospinal fluid (CSF) evaluation, myelography, and magnetic resonance imaging (MRI). MRI is the preferred antemortem method for diagnosing FCE because its findings may reflect abnormalities compatible with spinal cord ischemia. However, in 21% of suspected FCE cases, normal MRI findings were reported, which shows the limitations of using conventional MRI (2). For this reason, it is a great challenge to diagnose and confirm FCE with only conventional MRI. To overcome these problems in humans, MRI with diffusion-weighted imaging (DWI) and apparent diffusion coefficient (ADC) maps are used in the acute phase of spinal cord ischemia to identify restricted diffusion caused by intracellular cytotoxic edema (3–5).

In veterinary medicine, MRI with DWI and ADC mapping has rarely been applied to the confirmation of cranial and spinal ischemic injury. Although the DWI and ADC mapping of cranial infarction have been reported in both dogs and cats (6–8), there are no reports of the DWI and ADC mapping of spinal ischemic injury in veterinary medicine. Therefore, this case report first demonstrates the use of DWI and ADC findings in a clinical case of a dog histologically diagnosed with FCE.

## Case Presentation

A 9-year-old, non-ambulatory intact male Shih Tzu dog presented to our clinic 1 day after the peracute onset of neurological signs. Upon physical examination, the dog weighed 6.5 kg, had a pulse rate of 86 per minute, a respiratory rate of 32 breaths per minute, and a rectal temperature of 38.4°C. On neurologic examination, a tetraplegia was identified with lower motor neuron (LMN) symptoms, including decreased to absent spinal reflexes, flaccid paralysis, and muscle atrophy, whereas his mental status was alert and all cranial nerve examinations were normal. Laboratory examinations did not show any remarkable abnormalities.

Initially, diseases that cause acute flaccid tetraparesis, such as acute fulminating myasthenia gravis, polyradiculoneuritis, tick paralysis, and botulism, were suspected. During hospitalization, electrophysiologic examinations and neostigmine challenging tests were performed to differentiate between neuromuscular diseases. Electromyography and repetitive motor nerve stimulation tests conducted to evaluate denervation of the muscle and peripheral motor system, especially on the quadricep femoris muscle, were both normal. In addition, when injected with neostigmine methylsulfate (0.04 mg/kg subcutaneously; Dai Han Pharm. Co., Ltd, Seoul, South Korea), which is an acetylcholinesterase inhibitor, no improvement was observed. Tick paralysis was ruled out because the dog had not visited an endemic area and an engorged tick was not found on his body. Furthermore, although we did not test for botulinal neurotoxin or botulinal neurotoxin-antibody titer, botulism was also excluded because of no exposure to spoiled food. However, polyradiculoneuritis could not be completely excluded because we could not rule out the idiopathic type.

**Figure 1:**
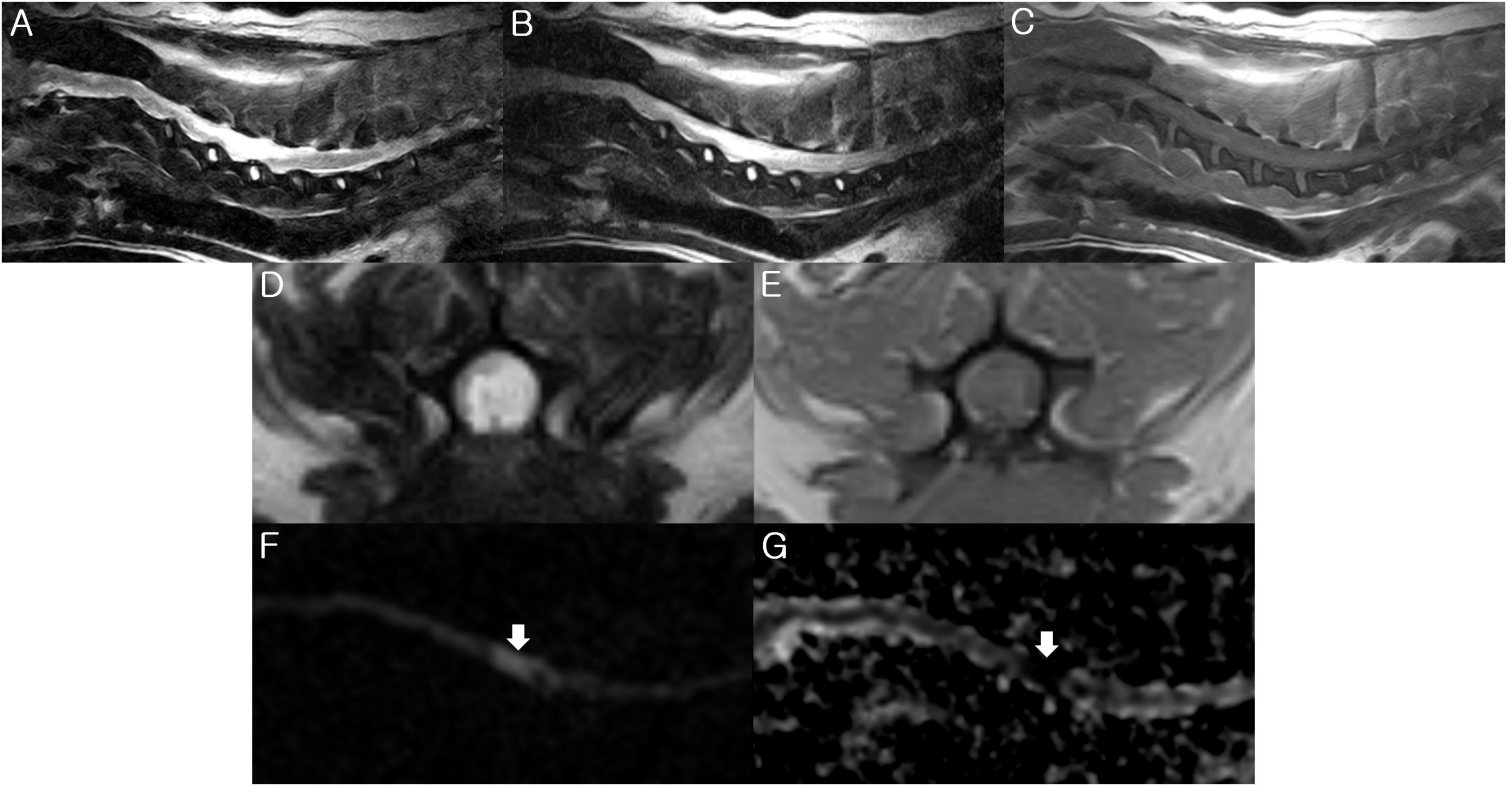
Magnetic resonance imaging (MRI) of the present dog. T2-weighted **(A,D)**, fluid-attenuated inversion recovery (FLAIR) **(B)**, and T1-weighted **(C,E)** images were obtained by 0.3-Tesla MRI 2 days after the initial onset of clinical signs. The diffusion weighted image (DWI) **(F)** and apparent diffusion coefficient (ADC) map **(G)** were taken by a 3-Tesla MRI 6 days after the initial onset of clinical signs. **(A,B)** There is hyperintensity in the T2-weighted and FLAIR images between the C3-C6 spinal cord parenchyma. Sagittal view. **(C)** There is isointensity in the T1-weighted image between the C3-C6 level. Sagittal view. **(D)** There is hyperintensity in the T2-weighted image at the C5 spinal cord parenchyma. Transverse view. **(E)** There is hypo- to iso- intensity in the T1-weighted image at the C5 spinal cord parenchyma. Transverse view. **(F)** High signal intensity is identified at the C5 spinal cord level (arrow) on the sagittal DWI. **(G)** ADC map shows low signal intensity at the C5 spinal cord (arrow). Sagittal view. These findings match the characteristics of FCE.

MRI examination of the spinal cord was performed using a 0.3-Tesla unit (Airis II, Hitachi, Japan) 2 days after the initial clinical signs. General anesthesia was induced with intravenous administration of propofol (6 mg/kg; Provive, Myungmoon Pharm. Co., Ltd, Seoul, South Korea) and maintained by inhalation of 2.0 to 2.5% isoflurane (Terrell, Piramal Critical Care, Bethlehem, PA, USA) in a circle rebreathing circuit. T1-weighted, T2-weighted, and fluid-attenuated inversion recovery (FLAIR) images were obtained using transverse, sagittal, and dorsal planes. The main lesion was identified between the C3-C6 spinal cord parenchyma through hyperintensity in the T2-weighted and FLAIR images, as well as hypointensity to isointensity in the T1-weighted images (Figures 1A–E). Additionally, mild to moderate compressions of the spinal cord parenchyma between the L1 and L5 level were also identified. After administration of gadolinium-diethylenetriamine pentaacetic acid (0.1 mmol/kg, IV; Omniscan™, GE Healthcare (Shanghai) Co., Ltd, China), post-contrast T1-weighted images were taken, but they exhibited no enhancement. For cerebrospinal fluid analysis, the total nucleated cell count was 465 cells/μL (reference range <5 cells/μL) with neutrophilic pleocytosis (neutrophil 91%, lymphocyte 7%, and monocyte 2%) and protein concentration was 30 mg/dL (reference range <25 mg/dL). Polymerase chain reactions for infectious agents were all negative (*Bartonella spp., Blastomyces dermatitidis, Coccidioides spp., Cryptococcus spp., Histoplasma capsulatum*, Canine distemper virus, West Nile virus, *Borrelia burgdorferi, Neospora spp., and Toxoplasma gondii*). Based on the medical history, signalment, clinical assessments, and laboratory findings, although bacterial myelitis was not completely ruled out due to the absence of a CSF culture, the dog was tentatively diagnosed with non-infectious myelitis or FCE with concurrent lumbar intervertebral disc disease (IVDD).

Initially, the dog was treated with prednisolone (1 mg/kg twice daily; Solondo®, Yuhan, Seoul, South Korea) and pentoxifylline (15 mg/kg twice daily; HARIN sustained-release Tab., Celltrion, Inc., Incheon, South Korea) immediately after MRI examination. On the third day of hospitalization, the dose of prednisolone was increased to 2 mg/kg twice daily. However, because there was a loss of deep pain perception in the left forelimb on the 4th day of hospitalization, we decided to reexamine with MRI using a 3-Tesla unit to identify the presence of an acute ischemic spinal disease, such as FCE, using DWI and ADC maps on the 6th day of hospitalization. Using an echo planar sequence, we acquired sagittal sliced DWI sections with b values of 0 and 1,000 s/mm^2^ along all three axes, with the following parameters: repetition time, 4,164 ms; echo time, 107 ms; flip angle, 90°; number of slices, 10; slice thickness, 3 mm; slice gap, 0 mm; field of view, 150 × 150 mm; and acquisition matrix, 140 × 140. A high signal intensity was identified between the C4-C6 level corresponding to the lesion observed on the previous MRI on the sagittal DWI (Figure 1F). A region of interest was manually drawn on the hyperintensity area of DWI and copied to the ADC map at the corresponding level. The ADC map was computed based on a pixel-by-pixel basis from the DWI. It was calculated using OsiriX MD v10.0 (Pixmeo Sarl, Geneva, Switzerland) and showed a low value (0.54 × 10^−3^ mm^2^/s; ADC value of normal spinal cord, 0.93 × 10^−3^ mm^2^/s) (Figure 1G). Based on these results of the 3-T MRI, in addition to the poor response to the immunosuppressive treatment, we strongly suspected the dog had FCE. On the 7th day of hospitalization, the dose of prednisolone was decreased to 1 mg/kg twice daily. The loss of deep pain perception in all the limbs was identified, suggesting a poor prognosis, and the dog suddenly died due to respiratory difficulty, suspected to be caused by ascending myelomalacia which led to intercostal and diaphragmatic paralysis on the 9th day of hospitalization (10 days after the initial clinical signs). The owner donated the dog for necropsy.

**Figure 2:**
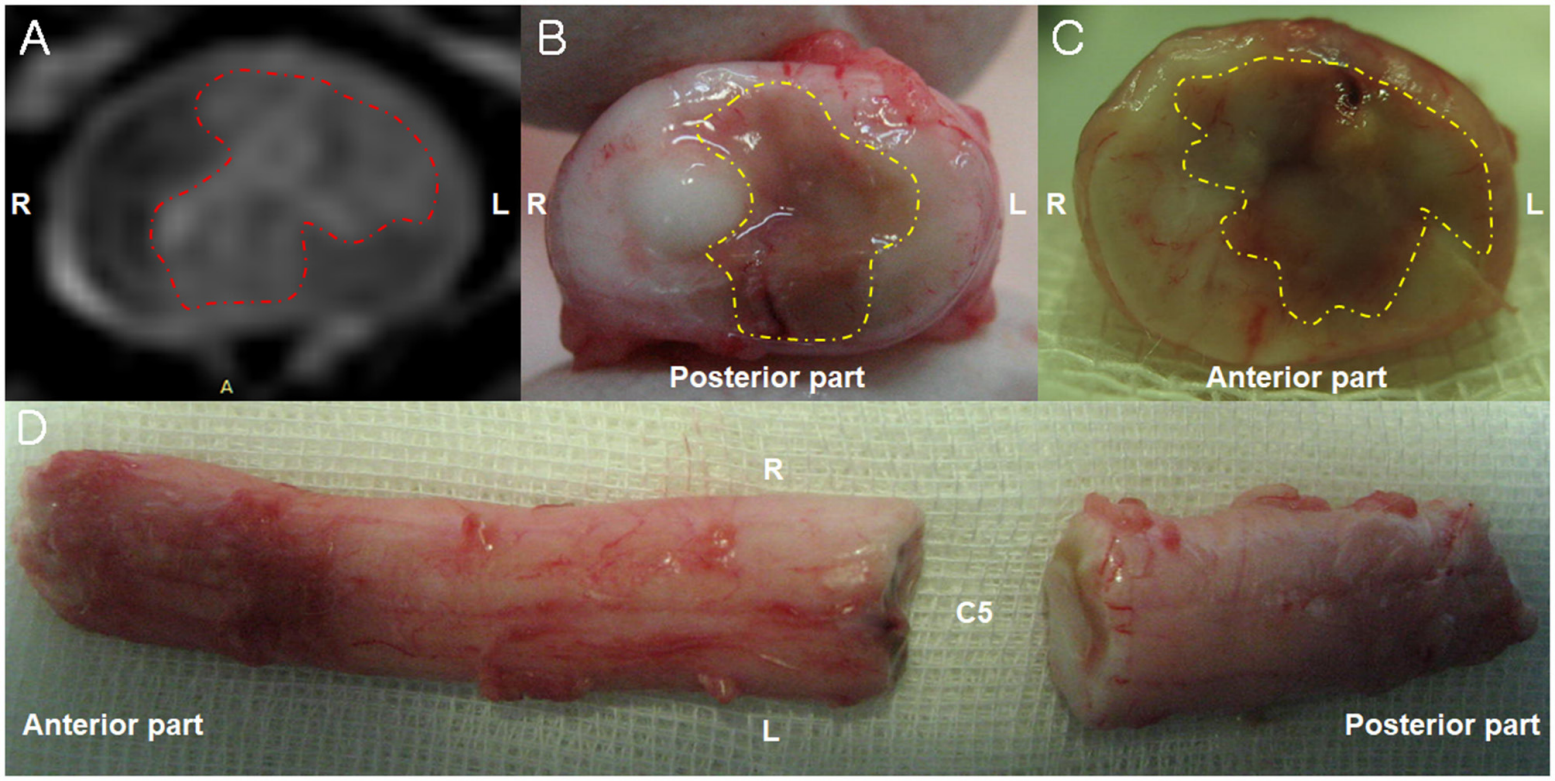
Magnetic resonance imaging (MRI) and necropsy findings of a fibrocartilaginous embolism in the cervical spinal cord. **(A)** There is hyperintensity (red dotted line) in the left spinal parenchyma in the T2-weighted image observed on a 3-Tesla MRI 6 days after the initial onset of clinical signs. Transverse view. **(B,C)** A necrotic area (yellow dotted line) is observed in left part of the spinal cord parenchyma 10 days after the initial onset of clinical signs. Transverse view. **(D)** The dorsal view of the cervical spinal cord 10 days after the initial onset of clinical signs.

**Figure 3:**
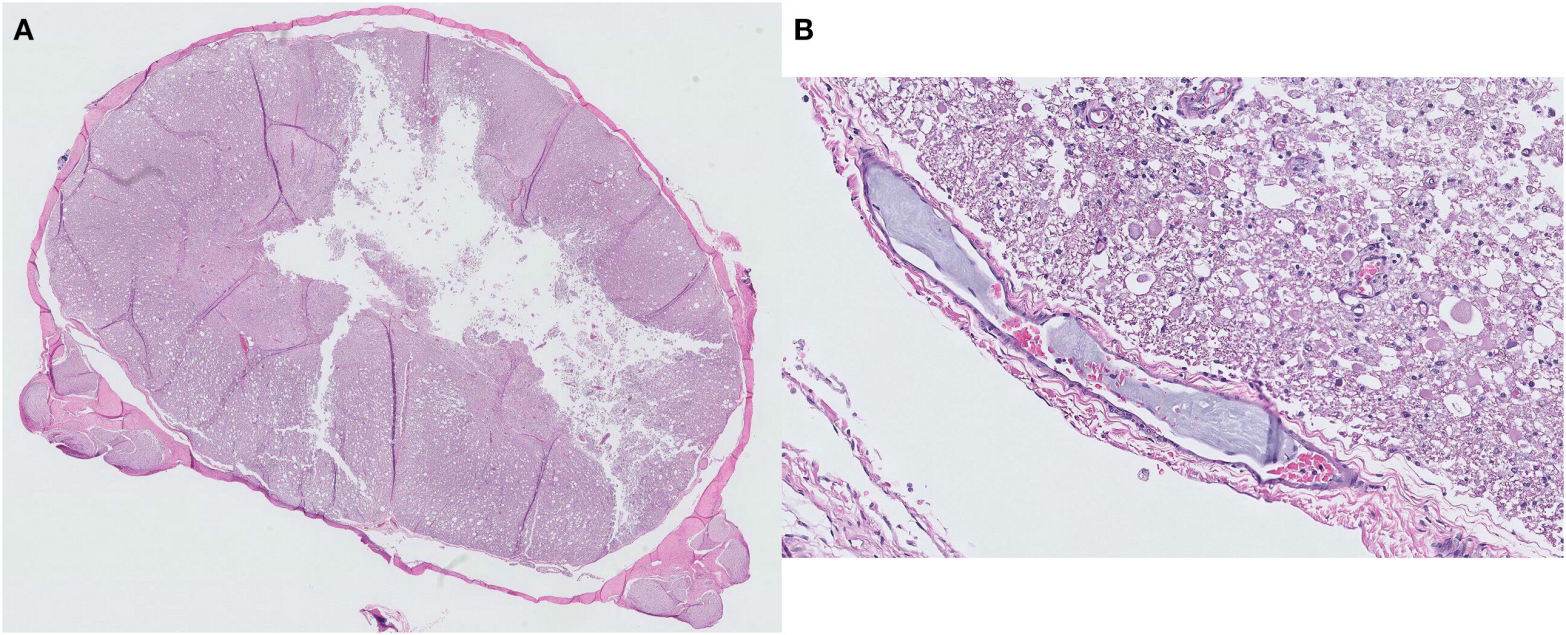
Histopathological findings of a fibrocartilaginous embolism at the C5 spinal cord level. **(A)** Liquefaction necrosis of the gray matter is observed with an almost total loss of the central gray column. Hematoxylin and eosin, ×5 magnification. **(B)** A small artery with a fibrocartilaginous embolus within its lumen. Hematoxylin and eosin, ×200 magnification.

Necropsy revealed necrotic regions in the area where the lesion was observed on MRI (Figure 2). Furthermore, a necrotic region was also found at the C2 level that was not identified through MRI. A histopathological examination revealed the presence of a fibrocartilaginous embolus in a small artery at the C5 level (Figure 3). There was also liquefaction necrosis of the gray matter with almost a total loss of the central gray column. Associated with this area of necrosis were accumulating numbers of Gitter cells with fewer neutrophils. In the surrounding white matter tracts, dorsally, laterally, and ventrally, there were variable numbers of degenerated and swollen axons. Similar lesions were present at both sites although marked at C5 and mild at C2. Based on these findings, the dog was definitively diagnosed with FCE presumed to have died due to ascending myelomalacia.

## Discussion

In the present case, a chondrodystrophic dog with tetraplegia was diagnosed with an ischemic spinal injury visualized by DWI and ADC map with high-field MRI, and FCE was definitively confirmed through histological examination.

Large and giant breed dogs are reportedly the most commonly predisposed to FCE, whereas small dogs with FCE have rarely been described (2, 9–11). More than 80% of dogs diagnosed with FCE are reportedly >20 kg in body weight (9). FCEs are generally reported in non-chondrodystrophic breed dogs, with only three confirmed reports in chondrodystrophic breed dogs (10–12). FCE have been described mainly in patients aged 5 or 6 years (range, 2 months to 11 years and 11 months) (2, 13, 14). In the current case, the dog was a 9-year-old chondrodystrophic toy breed (Shih Tzu dog) weighing only 6.5 kg.

Dogs with FCE generally have a peracute onset of non-painful, non-progressive (after the first 24 h), and often asymmetric myelopathy. FCE is also known to be related to physical activity, such as walking, running, and playing, at the time of clinical onset (2, 9, 13). Clinical signs vary depending on the severity and location of the spinal cord ischemic injury. L4–S3 (44–50%) and T3–L3 (27–42%) areas of the spinal cord are the most commonly affected locations in dogs with FCE diagnosed antemortem (2, 9, 13). In dogs with a definitive diagnosis of FCE, identified by histopathologic examination, the most commonly affected spinal cord locations are L4–S3 (43–47%) and C6–T2 (30–33%) (9, 13). In this case, the dog had a peracute onset with non-painful clinical signs after physical activity, and the lesion was located at the C3–C6 level.

The dog died due to suspected ascending myelomalacia, which caused intercostal and diaphragmatic paralysis. The prognosis of FCE is associated with the size of the lesion as measured on MRI. A lesion length/vertebral (C6) length ratio (sagittal plane) >2 or a cross-sectional lesion area percentage (transverse plane) >67% predicts significantly unsuccessful outcomes (15). The lesion length/vertebral length ratio and cross-sectional lesion area percentage of the dog in the present case were 5.18 and 77%, respectively, indicating a bad prognosis.

The present case of FCE was diagnosed with a hyperintense signal on DWI and a hypointense signal on ADC mapping 6 days after the initial clinical signs. DWI, which is sensitive to water mobility, has been widely applied for the investigation of acute cerebral and spinal ischemic stroke in humans. DWI depends on reduced diffusion caused by cytotoxic edema within ischemic cells because of the restricted Brownian motion of water molecules (16–18). The ischemic core of a stroke is shown by DWI as a hyperintense signal within minutes of onset; therefore, DWI is highly sensitive for confirming ischemic stroke early, which may not be detected on conventional MRI (16, 19). ADC is a measure of the magnitude of the diffusion of water molecules within tissue, and is commonly clinically calculated using MRI with DWI. The average ADC value of human spinal cord ischemia is known to be about 0.65 × 10^−3^ mm^2^/s (5), and the ADC value of the present case was 0.54 × 10^−3^ mm^2^/s. The ADC value of normal spinal cord tissue in the present case was 0.93 × 10^−3^ mm^2^/s. Hyperintense DWI reflects a reduced ADC value of water in the ischemic region (20). Therefore, if a lesion has a high signal in DWI images with low ADC values, the lesion is likely to have restricted diffusion of water molecules.

Normal MRI findings have been reported in 21% of suspected FCE cases in dogs with use of conventional MRI (2). For this reason, DWI and ADC maps have been applied for the detection of early lesions in ischemic diseases in humans (3–5). In a human ischemic cerebral stroke (21), ADC mapping and DWI show low signal intensity and high signal intensity, respectively, in early hyperacute (0–6 h), late hyperacute (6–24 h), and acute (24 h-1 week) phases. In subacute (1–3 weeks) and chronic (>3 weeks) phases, a low signal intensity may occur for 7–10 days and a pseudonormalization may occur 10–15 days after stroke onset on ADC mapping. After 15 days from stroke onset, a high signal intensity is maintained. In subacute (1–3 weeks) and chronic (>3 weeks) phases on DWI, a high signal intensity may occur for 10–14 days after stroke onset and normalization may occur early in the chronic phase. Although the use of DWI and ADC maps to detect ischemic spinal cord diseases is much more limited in humans, the high signal intensity and decrease in ADC value were identified 1–9 days after the onset of symptoms in DWI and ADC mapping, respectively (5). Similar to results shown in humans, in a canine ischemic cerebral stroke model, the ischemic lesion demonstrated DWI hyperintensity 3 and 10 days after middle cerebral artery occlusion (22). In MRI findings of canine spinal cord infarction models, the ischemic lesions demonstrated DWI hyperintensity and decreased ADC values within an hour post-embolism (23). Although serial findings of DWI and ADC in canine ischemic spinal diseases for more than 1 day have not been reported, the results of the current case were similar to those of the human ischemic diseases (brain and spinal cord) and a canine ischemic brain model.

Myelitis and spinal cord infarction are rare diseases in humans, and the differentiation criteria between both disorders have rarely been reported. However, Kim et al. (24) reported that idiopathic acute transverse myelitis does not show restricted diffusion of water molecules, which could be a criterion to differentiate it from spinal cord infarction. Most patients with acute transverse myelitis showed iso-signal intensity on ADC and a high signal intensity on DWI. Specifically, among 16 human patients with acute transverse myelitis, 14 showed an iso-signal on the ADC map, except for a relatively larger extent of lesion involvement. Therefore, if a lesion has a high signal in DWI images with normal ADC values, the lesion is likely to be myelitis rather than ischemic spinal injuries.

The cause of the LMN symptoms of the hindlimbs was not confirmed. Although the IVDD (specifically at the L4-L5 level) might cause LMN symptoms in the hindlimbs, the level of compression in this present case was thought to be mild. Therefore, a peripheral nerve or muscle biopsy was necessary to differentiate neuromyopathy from other causes, including IVDD. In cases of spinal shock, differences between neuroanatomical localization and MRI lesions have been reported in ischemic spinal disease (2). Thus, discrepancies between clinical signs and MRI lesions should be considered when there is a severe spinal lesion. Although only one case of multiple ischemic lesion was reported in a dog with FCE (25), multiple lesion should also be considered as one of the causes.

The main limitations of this case are the fact that it is a single case, and that the alterations of DWI, ADC mapping, and conventional MRI over time were not investigated. The main advantage of DWI and ADC maps is in the early detection of ischemic damage in patients with a normal conventional MRI. Therefore, further studies using a larger number of dogs with histopathologically diagnosed FCE are necessary to establish their sensitivity. Moreover, DWI and ADC maps of other diseases, such as meningomyelitis, hemorrhage, and intramedullary tumor, should be investigated to determine the specificity in differentiating other causes of myelopathy.

To the best of our knowledge, this is the first reported case of an ischemic spinal injury visualized by DWI and ADC mapping with high-field MRI in a chondrodystrophic dog with FCE. Furthermore, DWI with an ADC mapping could be used to identify early ischemic spinal injury and differentiate ischemic lesions from other myelopathies as a diagnostic method in veterinary medicine in the future.

## Data Availability Statement

The original contributions presented in the study are included in the article/supplementary materials, further inquiries can be directed to the corresponding author/s.

## Ethics Statement

Ethical review and approval was not required for the animal study because the case report is a retrospective evaluation with no active interventional or research component. Written informed consent was obtained from the owners for the participation of their animals in this study.

## Author Contributions

TY, K-IL, YK, HK, M-PY, and B-TK contributed to management of the case. DC and CL was in charge of the MRI evaluation. TY and K-IL wrote the first draft of the manuscript. TY, YK, HK, DC, CL, M-PY, and B-TK participated in the revision of the manuscript. All authors read, commented on, and approved the final manuscript.

## Conflict of Interest

The authors declare that the research was conducted in the absence of any commercial or financial relationships that could be construed as a potential conflict of interest.
